# A five-step approach for developing and implementing a Rural Primary Health Care Model for Dementia: a community–academic partnership

**DOI:** 10.1017/S1463423618000968

**Published:** 2019-05-15

**Authors:** Debra Morgan, Julie Kosteniuk, Dallas Seitz, Megan E. O’Connell, Andrew Kirk, Norma J. Stewart, Jayna Holroyd-Leduc, Jean Daku, Tracy Hack, Faye Hoium, Deb Kennett-Russill, Kristen Sauter

**Affiliations:** 1 Professor and Chair of Rural Health Delivery, Canadian Centre for Health & Safety in Agriculture, University of Saskatchewan, Saskatoon, SK, Canada; 2 Professional Research Associate, Canadian Centre for Health & Safety in Agriculture, University of Saskatchewan, Saskatoon, SK, Canada; 3 Associate Professor, Department of Psychiatry, Queen’s University, Providence Care - Mental Health Services, Kingston, ON, Canada; 4 Associate Professor, Department of Psychology, University of Saskatchewan, Saskatoon, SK, Canada; 5 Professor, Department of Medicine, Head of Neurology, University of Saskatchewan, Saskatoon, SK, Canada; 6 Professor Emerita, College of Nursing, University of Saskatchewan, Saskatoon, SK, Canada; 7 Section Chief, BSF Chair in Geriatric Medicine and Professor, Section of Geriatrics, Departments of Medicine and Community Health Sciences, University of Calgary, Alberta, Canada; 8 Nurse Practitioner, Sun Country Health Region Kipling, Saskatchewan, Canada; 9 Home Care Nurse, Sun Country Health Region, Kipling, Saskatchewan, Canada; 10 Business Manager, Primary Health Care, Sun Country Health Region, Kipling, Saskatchewan, Canada; 11 Occupational Therapist, Regional Manager of Therapies, Sun Country Health Region, Kipling, Saskatchewan, Canada; 12 Facilitator, Primary Health Care, Sun Country Health Region, Kipling, Saskatchewan, Canada

**Keywords:** community-based participatory research, dementia, implementation, primary health care, rural

## Abstract

**Aim:**

This study is aimed at developing a Rural Primary Health Care (PHC) Model for delivering comprehensive PHC for dementia in rural settings and addressing the gap in knowledge about disseminating and implementing evidence-based dementia care in a rural PHC context.

**Background:**

Limited access to specialists and services in rural areas leads to increased responsibility for dementia diagnosis and management in PHC, yet a gap exists in evidence-based best practices for rural dementia care.

**Methods:**

Elements of the Rural PHC Model for Dementia were based on seven principles of effective PHC for dementia identified from published research and organized into three domains: team-based care, decision support, and specialist-to-provider support. Since 2013 the researchers have collaborated with a rural PHC team in a community of 1000 people in the Canadian province of Saskatchewan to operationalize these elements in ways that were feasible in the local context. The five-step approach included: building relationships; conducting a problem analysis/needs assessment; identifying core and adaptable elements of a decision support tool embedded in the model and resolving applicability issues; implementing and adapting the intervention with local stakeholders; and sustaining the model while incrementally scaling up.

**Results:**

Developing and sustaining relationships at regional and PHC team levels was critical. A comprehensive needs assessment identified challenges related to all domains of the Rural PHC Model. An existing decision support tool for dementia diagnosis and management was adapted and embedded in the team’s electronic medical record. Strategies for operationalizing other model elements included integrating team-based care co-ordination into the decision support tool and family-centered case conferences. Research team specialists provided educational sessions on topics identified by the PHC team. This paper provides an example of a community-based process for adapting evidence-based practice principles to a real-world setting.

An estimated 47 million people worldwide are living with dementia [Alzheimer’s Disease International (ADI), 2015], a prevalence that is projected to double every 20 years. A large proportion of those with dementia live in low resource rural/remote (‘rural’) settings, thus there is a critical need to design effective and sustainable dementia care strategies for these settings (Innes *et al*., 2011; Morgan *et al*., 2011; Morgan *et al*., 2015; ADI, 2015; 2016). Rural areas have a higher proportion of seniors compared to urban areas (Elliot, 2012) due to out-migration of younger and in-migration of older adults (Milbourne, 2012), and increasing dementia risk with age. Yet there are numerous barriers to high-quality dementia care in rural areas including limited services and specialist access (Morgan *et al*., 2011; Szymcynska *et al*., 2011; Greenway-Crombie *et al*., 2012; Morgan *et al*., 2012; Dal Bello-Haas *et al*., 2014; Innes *et al*., 2014; Kosteniuk *et al*., 2014b; Morgan *et al*., 2014a; 2014b; O’Connell *et al*., 2014; Auer *et al*., 2015) leading to increased responsibility for dementia diagnosis and management in rural primary health care (PHC).

Addressing barriers to dementia care in low resource settings and addressing geographic inequalities in care was a priority by the G8 Global Dementia Group (ADI, 2015). A comprehensive, holistic, and integrated PHC approach to dementia care is widely accepted as best meeting the needs of people with dementia and their caregivers (Callahan *et al*., 2006; Vickrey *et al*., 2006; Aminzadeh *et al*., 2012; World Health Organization and Alzheimer’s Disease International, 2012). The 2016 World Alzheimer Report (ADI, 2016) notes that models of health service delivery in high income countries tend to be specialized, with little formal recognition of the role of PHC. Limitations of this specialist model include under-resourced specialist services and inability to facilitate care co-ordination, a key function of PHC. Thus an alternate model with a central role for PHC is recommended, especially for resource-poor settings (ADI, 2016). In rural areas with limited access to services, the scope of primary care providers’ practice in dementia care will need to be broader, but the roles and responsibilities of team members have not been fully explored (Pond, 2017).

Current models of PHC for dementia incorporate key components of comprehensive care (Bergman, 2009; Lee *et al*., 2010; Callahan *et al*., 2011; Engeda *et al.*, 2012) but do not specifically account for rural contextual factors, including poor specialist access, few PHC team members (often not co-located), small group practices, and few dementia-specific resources (Szymcynska *et al*., 2011; Greenway-Crombie *et al*., 2012; Innes *et al*., 2014). Not only are rural settings different from urban; there is also tremendous diversity across rural settings and PHC teams. Thus, best practices must be tailored to community needs and resources and be sustainable while preserving effectiveness (Aarons *et al*., 2012; Cabassa *et al*., 2014). Collaborative community-based participatory research approaches emphasize co-production of knowledge within the context where it is used, thereby increasing relevance and use of the evidence to practice communities (Rycroft-Malone *et al*., 2016). Community-based participatory research begins with assessing community priorities and then collaboratively developing or adapting the intervention (Wallerstein and Duran, 2010). Ritchie *et al*. (2013) noted that few studies have explored ways of implementing these principles when partners are located in rural or remote communities distant from research centers.

## The rural dementia action research (RaDAR) program

For two decades the RaDAR team has conducted a community-based participatory program aimed at improving health service delivery for people with dementia and their families in rural and remote settings (Morgan *et al*., 2014a) including the development of a telehealth-supported university-based rural and remote memory clinic in 2004 (Morgan *et al*., 2009; O’Connell *et al*., 2014). The principles that inform our research include ongoing two-way exchange between community members and researchers to understand each other’s needs and priorities, use of research data to inform action for community benefit, incorporating local knowledge developing interventions to ensure applicability and community fit, and improving research and program development capacity of communities (Israel *et al*., 1998; Shalowitz *et al*., 2009). Our research has identified challenges in dementia assessment, diagnosis, and post-diagnostic support in rural settings. Rural dementia-specific services are mostly unavailable and many existing services are inadequate (Innes *et al*., 2011; Morgan *et al*., 2011; 2015; Kosteniuk *et al*., 2016b).

Although rural health research has had a predominant focus on problems and deficits, and our own research has identified challenges in provision of care for people with dementia, we adhere to a strengths-based and problem-solving perspective that recognizes that rural communities are often sites of innovation in designing models of health care for the local context (Bourke *et al*., 2010). A strengths-based approach encourages community participation to improve what works and address shortcomings. Rural health care providers often have greater knowledge of the community and their patients than external researchers, and thus a better understanding of local health challenges and potential solutions (Bourke *et al*., 2010).

The RaDAR team’s focus on rural PHC was developed in response to the needs identified in our research and that of others, and issues observed in providing specialist care in our memory clinic. A recent scoping review (Lourida *et al*., 2017) identified a gap in evidence to inform dissemination and implementation of evidence-based dementia care, especially in PHC settings. The review authors concluded that there is an urgent need for more attention and resources for dissemination and implementation research in dementia care to support practice change. In Canada, a key message from a 2015 national forum on dementia was the lack of scale-up mechanisms and funding to ensure translation of research and best practices to all communities (Feldman and Estabrooks, 2017).

The RaDAR team developed the Rural PHC Model for Dementia, deriving the elements from an extensive scoping review conducted outside of the team. The review synthesized and critically analyzed a decade of international literature on barriers and enablers to timely diagnosis and optimal management of community-living persons with dementia in PHC (Aminzadeh *et al*., 2012). The review found that models of community-based dementia care that were more integrated and provided more comprehensive dementia-specific services in PHC were associated with better outcomes. These intensive interventions incorporated a combination of seven key elements, or best practice principles, which we organized into three domains to provide the foundation for the Rural PHC Model for Dementia: (1) *team-based care* (multidisciplinary team; ongoing care management; education and support for patients and caregivers); (2) *decision support tools* (access to standardized tools, guidelines, and protocols; access to information technology resources such as electronic medical records (EMRs)); and (3) remote *specialist-to-provider support* (access to dementia specialists; provision of formal dementia training to PHC providers).

The long-term goal of the research reported here was to develop feasible and effective approaches for operationalizing each element of the model, beginning with one rural PHC team and then incrementally scaling up to build an inventory of strategies for implementing each model element. The inventory will be adaptable, scalable, and sustainable across diverse rural settings and PHC teams. The aim of this paper is to describe the development, adaptation, and implementation phases of a Rural PHC Model for Dementia designed to address the gap in dementia care best practices for PHC in rural settings. This paper provides an example of a process for operationalizing and adapting evidence-based practice principles to a real-world setting.

## Methods

This study was conducted in the western Canadian province of Saskatchewan (population 1098352, area 651 035 km^2^, 1.9 persons/km^2^), where 39% of the population lives in rural areas with less than 10 000 population (Moazzami, 2015) compared to 19% nationally. At the time of the study, Saskatchewan had 13 health regions with authority to allocate health service resources in the region. We partnered with Sun Country Health Region (33 239 km^2^, population 59 984, 1.8 persons/km^2^, 15% of population ⩾ age 65) in southeast Saskatchewan. The region has two cities (populations 10 679 and 11 258) with the remaining 58% of the population residing in rural communities. Seven PHC teams are spread across the region, with most specialist referrals requiring travel to the city of Regina, located at least 1.5 h from most communities.

This paper describes our collaboration with one of the PHC teams in the region (Team 1), located in a town of just over 1000 people, ~400 km from the University of Saskatchewan in Saskatoon where most members of the RaDAR team are located. Team 1 was established in 2013, just before we began working with them, and consists of seven health care providers (three family physicians, a nurse practitioner, an occupational therapist, and two home care nurses). The project received ethics approval from the University of Saskatchewan Behavioral Research Ethics Board and operational approval from Sun Country Health Region.

### Development–adaptation approach

Our goal in this developmental study was to begin to put into practice the principles of effective PHC for dementia in the Rural PHC Model, by developing strategies aligned with these principles that would be feasible, effective, and sustainable in rural settings. Embedded in this development work was an adaptation of the Primary Care – Dementia Assessment and Treatment Algorithm (PC-DATA^TM^) (Seitz, 2012), a set of existing decision support tools based on the most recent Canadian consensus guidelines on dementia (Moore *et al*., 2014). The five-step approach used in the current study was informed by four published frameworks for modifying evidence-based interventions for local settings (McKleroy *et al*., 2006; Lee, 2008; Jansen *et al*., 2013; Cabassa *et al*., 2014).

Table 1 describes the activities undertaken at each step. The activities in Step 1 that contributed to building relationships at the regional level included forming a steering committee, conducting a regional dementia learning needs assessment with 82 home care providers (Kosteniuk *et al*., 2014a; 2016a; Morgan *et al*., 2016), and publishing regular newsletters with study updates. Team 1 also engaged in relationship-building activities by taking part in teleconferences and educational sessions related to the intervention, and attending events with RaDAR team members. At Step 2, the problem analysis and needs assessment stage, we conducted a provincial gap analysis that captured regional-level data (Kosteniuk *et al*., 2015b), as well as a regional baseline needs assessment with 32 participants, including members of seven PHC teams across the region (*N*=19), regional decision-makers (*N*=9), and patients and caregivers (*N*=4) (Kosteniuk *et al*., 2016b). Needs assessment at the PHC team level drew on Team 1 data collected in the regional baseline needs assessment and a pre-implementation focus group with Team 1 members and regional decision-makers (*N*=12). During Step 3, the main activities related to intervention assessment focused on identifying core and adaptable elements of a decision support tool (PC-DATA^TM^) and resolving applicability issues with support from the tool developer and five other dementia experts and researchers. Step 4 activities to support iterative implementation and adaptation of the Rural PHC Model for Dementia intervention involved one information session and four education sessions, four focus groups with Team 1, and eight meetings with the Team 1 working group and support staff. During Step 5, we held six meetings with the working group from Team 1 to sustain the intervention in their PHC site, and simultaneously began to scale-up incrementally to other PHC sites by launching the five-step approach with PHC Team 2 in 2017. A process evaluation informed by the Consolidated Framework for Implementation Research (Damschroder *et al*., 2009), to be reported elsewhere, was conducted to evaluate the contextual factors that influenced the development-adaptation process across the five steps with Team 1.

**Table 1:** Five-step development-adaptation process with activities and outcomes

Step	Activities	Outcomes
1. Build relationships	**Regional Level** Regional director membership in RaDAR Team Decision Maker Advisory Council since 2008 Regional Steering Committee formed 2013 (regional leadership, RaDAR, Alzheimer Society), Terms of Reference developed; quarterly meetings held RaDAR conducted a dementia learning needs assessment of 82 home care providers to inform regional dementia education programs and services Quarterly RaDAR newsletters with study updates and RaDAR research and knowledge exchange activities **Primary Health Care Team Level** Working Group (subgroup of PHC Team 1) established Meetings and teleconferences with full Team 1 and Team 1 Working Group RaDAR provided educational sessions Team members attended memory clinic led by researchers, attended national research conferences, participated in annual RaDAR Rural Dementia Summit	Regional Level Five-year project commitment from Sun Country Health Region; regional resources assigned to project Access to regional staff promoted Region assisted with recruitment for Baseline Needs Assessment study PHC Team 1 was identified by the regional Steering Committee to develop initial working model Primary Health Care Team Level PHC Team 1 agreed to participate in the study and commit time to the project, and has remained engaged
2. Problem analysis and needs assessment	**Regional Level** RaDAR conducted a provincial dementia care Gap Analysis (report included regional data on dementia incidence, prevalence, service availability) RaDAR conducted a Regional Baseline Needs Assessment (*N*=32) and provided report to health region **Primary Health Care Team Level** Regional Baseline Needs Assessment included Team 1 Pre-implementation focus group held with Team 1 to assess needs Ongoing informal needs assessment is built into implementation process	Regional Level Provincial Gap Analysis found that SCHR had the highest age- and sex-adjusted dementia incidence rate and second highest prevalence rate across 13 SK regions, and low availability of dementia-related services Regional Baseline Needs Assessment identified best practice gaps and strengths in PHC for dementia; guided development of a tailored intervention to address gaps Primary Health Care Team Level Needs assessment identified key areas for intervention development to address local needs
3. Assess elements of intervention to be adapted; resolve applicability issues with program developer and experts	Literature reviewed to identify key elements of effective PHC for dementia Identification of an existing decision support tool (PC-DATA) that addressed identified needs and operationalized the Rural PHC model element of ‘access to standardized tools and guidelines’ Analyzed original PC-DATA^TM^ tool (visit flow sheets, algorithms, education manual) to itemize key elements Held face-to-face meetings and teleconferences with PC-DATA^TM^ developer and five other dementia experts and researchers to identify core and adaptable program elements	Rural Primary Health Care Model for Dementia developed based on seven key elements of effective PHC for dementia identified in the literature (Aminzadeh): multidisciplinary team, ongoing care management, education/support for patients and caregivers, access to standardized tools and guidelines, access to IT resources, access to dementia specialists, formal dementia training for PHC providers Core and adaptable elements of the PC-DATA^TM^ decision support tools were identified and modified based on program developer and expert analysis to fit local health system
4. Implement intervention and resolve applicability issues with PHC team (ongoing adaptation)	Information session with Team 1 to review the Rural PHC Model Education sessions to Team 1 by dementia specialists on research team Team 1 implementation and post-implementation focus groups Meetings with Team 1 Working Group and EMR support staff Iterative testing and adaptations of Rural PHC Model elements	Co-development of strategies to operationalize core principles in the Rural PHC Model, to create the first iteration of the Rural PHC Model RaDAR Handbook Team 1 Home care nurse given access to EMR to enable participation in study Creation of interdisciplinary EMR visit flow sheet by regional EMR manager, from adapted paper-based PC-DATA^TM^ visit flow sheets
5. Sustain the intervention in initial PHC team site while scaling up to other sites	Ongoing meetings with Team 1 Working Group to support continued refinement of model elements and assess sustainability needs and issues Steps 1–5 repeated with Team 2 and Team 3 in different locations in the health region (different population size, team configuration, number of communities served by the team)	First iteration of Rural PHC Handbook developed by Team 1 was implemented and adapted by Team 2 and Team 3 Develop a network of PHC teams that have implemented the Rural PHC Model, to share adaptations and build an inventory/toolkit of rural best practices

RaDAR=Rural Dementia Action Research Team; PHC=primary health care; PC-DATA=Primary Care – Dementia Assessment & Treatment Algorithm^TM^; EMR=electronic medical record; SCHR=Sun Country Health Region; SK=province of Saskatchewan.

## Results

The activities and outcomes at each step in the five-step development–adaptation approach are summarized in Table 1 and described below in further detail.

### Step 1: building relationships

Engaging community partners is an essential first step in community-based participatory research, where the goal is enhancing the relevance, application, and sustainability of improvements in health care (Seifer, 2006). Co-production of locally applicable knowledge requires genuine collaboration and is dependent on the quality of relationships among stakeholders (Rycroft-Malone *et al*., 2016). In the current study, we developed relationships first between the RaDAR team and stakeholders at the health region level, and then with health care providers and staff at the PHC team level.

#### Regional level

Our connection with the Sun Country Health Region started in 2008 when the regional director of home care began attending the RaDAR team’s annual knowledge exchange event, the Knowledge Network in Rural and Remote Dementia Care Summit. In 2011 Sun Country’s directors of home care and PHC participated in a RaDAR meeting of provincial stakeholders and researchers to plan research priorities for a study focusing on rural PHC for dementia. The region provided a letter of support, indicating their interest in participating. Although the grant was not funded the region remained committed and established a formal 12-month initiative to improve the dementia knowledge base of Sun Country’s health care providers. A tripartite regional steering committee was formed in April 2013 to support this initiative and RaDAR team members (D.M., J.K.) were invited to join to provide dementia care and research expertise. The steering committee consists of 12–15 individuals, with membership fluctuating over time with retirements and the addition of new staff. The committee includes RaDAR team members, leadership from Sun Country’s sectors of home care, PHC, long-term care, social work, and chronic disease management, as well as the Alzheimer Society of Saskatchewan.

The RaDAR team was also committed to supporting the collaboration with the region while seeking project funding. We conducted a dementia learning needs assessment of home care providers (Kosteniuk, *et al*., 2014a; 2016a; Morgan *et al*., 2016) and supported the steering committee in developing and delivering an educational workshop for home care staff. The steering committee continued to meet after the educational initiative ended and now performs the dual function of regional dementia steering committee and advisory committee for the RaDAR research program. The committee meets quarterly by teleconference or in person to bridge gaps in dementia care in the region, support Alzheimer Society activities, and provide support to the RaDAR research program. To date the committee has met 21 times. As a result of this step, the region committed to the current five-year intervention project, provided resources, and promoted project participation by PHC teams and staff.

#### PHC team level

An important regional level outcome was identification of the first PHC team to participate in the study, based on the criteria of at least one dementia care champion and all team members willing to commit time to the study. Following an introductory meeting in August 2014, Team 1 agreed to partner with the RaDAR team. Team 1 established a working group of the team’s nurse practitioner, occupational therapist, a home care nurse, PHC facilitator, regional Alzheimer Society First Link Coordinator, and regional business manager who manages the EMR system used by the region’s seven PHC teams. Periodic meetings of the research team and the full PHC team (five meetings to date), and more frequent meetings with the smaller working group (14 meetings), have been key to ongoing relationship-building. A quarterly RaDAR newsletter was launched in 2014 as a knowledge exchange and communication tool and strategy for showcasing the progress being made by Team 1 in improving dementia care. Relationship building was also enhanced by providing travel funding for Team 1 to attend the RaDAR team’s Rural and Remote Memory Clinic, co-present at national dementia conferences, and present their perspectives of the collaboration at the annual RaDAR knowledge exchange Summit. Team 1 has been engaged in the intervention since 2014, the main outcome of this step at the PHC team level.

### Step 2: problem analysis and needs assessment

Local needs assessment is essential for identifying needs, strengths, and key areas for intervention adaptation (McKleroy *et al*., 2006). McKleroy *et al*. (2006) recommend assessment of the target population, intervention possibilities and goodness of fit with the population, stakeholder needs and potential for collaboration, and organizational capacity. In the current study, we conducted a broad needs assessment using three procedures, namely a provincial gap analysis study, a regional baseline needs assessment, and a pre-implementation focus group with Team 1. The main outcome of this step was the identification of priorities at the regional and PHC team levels that were integrated into the tailored intervention. Ethics approval was received from the University of Saskatchewan for all needs assessment studies.

#### Provincial gap analysis study

This study included three components: (1) an analysis of best practices across nine national dementia plans, (2) analysis of linked administrative health databases to determine incidence and prevalence of dementia among individuals aged 45 and older, and (3) an environmental scan of home care assessors to identify available dementia-related services and resources, and the orientation of these services toward key dimensions of PHC. Provincial level data on dementia incidence and prevalence (Kosteniuk *et al*., 2015a) and the environmental scan (Morgan *et al*., 2015) were previously published. A detailed report of methods and results by each of the 13 health regions in Saskatchewan is available (Kosteniuk *et al*., 2015b).

The main outcome of the provincial gap analysis was the finding that the Sun Country Health region had the highest age and sex-adjusted incidence rate (8.77 per 1000 population at risk) and the second highest adjusted prevalence rate (30.55 per 1000 population at risk) in the province. Further, the environmental scan found that the availability of dementia-related services varied by type of service. Services that were more available included access to most PHC providers (except nurse practitioners) and home care (except night, weekend, and emergency respite). Services that were mostly unavailable included health promotion, post-diagnostic support, and counseling for individuals with dementia and caregivers. Most ratings of how existing services were oriented to PHC principles were somewhat negative or neutral (information and education, accessibility, fit with community need, co-ordination, comprehensiveness, quality of care), with slightly positive ratings for co-ordination and comprehensiveness of care (Kosteniuk *et al*., 2015b).

#### Regional baseline needs assessment

We conducted a baseline study of dementia care best practice gaps and strengths in the Sun Country Health Region in 2015 before beginning the adaptation work with Team 1. A detailed report with study methods and findings is available (Kosteniuk *et al*., 2016b). Data were collected by telephone interview using structured questionnaires tailored for each of the three groups, consisting of 32 participants overall: regional decision makers (*N*=9), patients/caregivers (*N*=4), and health care providers from the seven PHC teams (*N*=19). Health region decision makers (regional steering committee and PHC team facilitators) reported that difficulty establishing a dementia diagnosis was the top regional challenge. Patients and caregivers described care from their PHC team members as personal, attentive, and informative. PHC team members, including Team 1 members, were asked whether the elements of the Rural PHC Model were present in their team on a 5-point scale from strongly disagree (1) to strongly agree (5). Mean scores were highest for team-based care, followed by access to decision support tools, and lowest for access to specialist-to-provider support. PHC team members also used a 5-point scale to rate overall team effectiveness in dementia assessment (*M*=3.6, SD=0.9), diagnosis (*M*=3.5, SD=1.1), and management (*M*=3.8, SD=0.9). The most frequent qualitative responses from PHC team members regarding what was most needed to improve dementia care were implementation of standardized processes for diagnosis and management, improved capacity for local diagnosis and management, improved specialist access, and earlier diagnosis.

#### Focus group with PHC Team 1

A pre-implementation focus group was held in July 2015 with seven Team 1 members (physicians, nurse practitioner, occupational therapist, home care nurses) and five regional directors/managers (community services, PHC teams, chronic disease management, mental health) to discuss current dementia care strengths and needs. Providers reported that they needed access to standardized tools and guidelines to help with assessment, diagnosis, and ongoing management issues such as driving. Lack of early diagnoses resulted in lost opportunity to introduce early support and education for patients and caregivers, and consequent crises resulting in long-term care placement. Barriers to improving care included lack of access to the EMR by some team members; absence of a case manager role; difficulty accessing dementia specialists and education programs; stigma; and resistance to assessment, diagnosis, and use of support services by patients and families. Strengths included multiple disciplines available to work together, access to Alzheimer Society resources and staff, and recent implementation of a shared EMR system.

Overall, outcomes from the multiple needs assessment approaches were consistent in identifying challenges related to all three domains of the Rural PHC Model and provided a strong rationale for developing rural-specific strategies for implementing model elements. Lack of access to evidence-based standardized tools and guidelines for dementia care emerged as a major barrier to early diagnosis and management.

### Step 3: assess elements of a decision support tool to be adapted and resolve applicability issues with tool developer and specialists (initial adaptation)

This study involved the development of rural-specific strategies to operationalize known best practices in PHC for dementia, as well as the adaptation of an existing decision support tool to the rural context. A brief description of the strategies that were developed for each element of the Rural PHC Model is provided below and summarized in Table 2.

**Table 2:** Rural Dementia Action Research Team (RaDAR) primary health care (PHC) model for dementia

Team-based care	Specialist-to-provider support	Decision support tools
Multidisciplinary team	Access to dementia specialists	Standard tools & guidelines
Family physician or nurse practitioner Home care nurse Occupational therapist Others such as office staff, PHC facilitator	Referral – to rural and remote memory clinic Consultation – with RaDAR specialists, PC-DATA developer Team 1 nurse practitioner, occupational therapist, and home care nurse observed rural and remote memory clinic team functioning	PC-DATA tools (visit flow sheets, education manual, and care pathways/algorithms). Three original visit flow sheets adapted and added to the regional EMR system as two flow sheets (initial assessment, and follow-up) with a separate section for each provider Work standards to guide the use of the EMR visit flow sheet and dementia case conferences were developed by PHC Team 1 Scripts for driving and communicating a diagnosis were developed by PHC Team 1
Care management	Education sessions	Access to IT resources
Team co-ordination has been built into EMR visit flow sheet Dementia case conferences implemented (between team and patient/family)	PC-DATA^TM^ info session PC-DATA^TM^ education sessions Differential diagnosis Capacity and competency Driving and dementia	All providers have access to work standards All providers have access to EMR visit flow sheet
Education/support for patient and caregiver		
Alzheimer Society First Link Coordinator is included in case conferences A link to Alzheimer Society referral form added to EMR flow sheet		

PC-DATA=Primary Care – Dementia Assessment & Treatment Algorithm^TM^; EMR=electronic medical record.

During the pre-implementation focus group, initial strategies were planned for operationalizing team-based care and specialist-to-provider support, which were implemented in Step 4. The RaDAR team was aware of existing PHC decision support tools developed and pilot tested in Ontario, based on Canadian consensus guidelines (Moore *et al*., 2014). The PC-DATA (Seitz, 2012) includes visit flow sheets, decision algorithms, and education materials. Team 1 was enthusiastic about adapting these tools for their use, thus the intervention included both adaptation work and development of new strategies for operationalizing the rural PHC model elements.

Adaptation of evidence-based interventions to fit a new situation can involve modification of the intervention components (eg, augmenting, emphasizing/de-emphasizing, cultural adaptations) and adapting aspects of the context (Aarons *et al*., 2012). A systematic approach to adaptation is essential to maintain fidelity to core components necessary for program effectiveness, while allowing for customization of adaptable or secondary components to the local context (Misfeldt *et al*., 2017). A detailed list of all PC-DATA components was reviewed by the program developer (D.S.) and five other clinical experts and researchers to assess their clinical relevance and applicability to the Saskatchewan rural context. Using a consensus process, Step 3 resulted in the identification of core and adaptable elements and suggestions for adaptations. Core elements included the initial information session/needs assessment meeting with the PHC team, initial and follow-up PC-DATA education and implementation sessions, education slides and manual, three patient visit flow sheets (assessment, diagnosis and initial management, ongoing assessment and monitoring) and algorithms (care pathways). Adaptable elements included role of the dementia care manager, some flow sheet content (eg, specific tools for cognitive and functional assessment), and delivery mode (eg, in-person versus telehealth).

### Step 4: implement and adapt intervention with the PHC team (ongoing adaptation)

Implementation and adaptation must be active processes that integrate the community partners throughout to make the innovation more relevant and increase ownership (McKleroy *et al*., 2006). Capacity training for practitioners, through training and ongoing support, helps to sustain the implementation (Lee *et al*., 2010; Wallerstein and Duran, 2010). To begin the development–adaptation process with Team 1, the PC-DATA developer provided an interactive education session to review the first visit flow sheet (assessment). Over 13 months, five focus groups were held with the full Team 1 and six meetings were held with the Team 1 working group and EMR support staff. RaDAR team specialists provided education sessions on topics identified by Team 1, including differential diagnosis and assessing capacity.

In an iterative process of adapting and testing, Step 4 resulted in operationalization of the three components of the Rural PHC Dementia model described below: team-based care, decision support, and remote specialist-to-provider support. These components were collated into the RaDAR Handbook to provider a resource for current team members and a tool for scaling-up to future teams. Regular meetings of the researchers with the full PHC team and working group were critical to development/adaptation work. Practices to activate the element of team-based care were developed and incorporated into an adapted PC-DATA visit flow sheet (eg, identifying individual team member roles in assessment) and team processes were modified (eg, case conferences implemented following assessment). Iterative testing of the flow sheet identified limitations that were addressed at subsequent meetings and further modifications worked out together.

### Team-based care

#### Multidisciplinary team

A strength of Team 1 was the availability of team members from different disciplines, but there were no formal processes for collaborating and co-ordinating care for individuals with dementia. The PC-DATA developer created the original flow sheets for primary care physicians supported by dementia care managers in the province of Ontario, and did not differentiate roles for other PHC team members. Team 1 identified that the paper-based PC-DATA flow sheets needed to be transferred into the EMR, which was used for all patient visits and accessible to most team members. The regional operations manager, who was also a member of the Team 1 working group, created EMR versions of the three original flow sheets. The EMR-based adapted flow sheet for assessment (flow sheet 1) was tested individually by a Team 1 physician and the nurse practitioner and found to be overly time-consuming. To distribute the assessment tasks and build in a team-based approach, team members identified sections of the flow sheet that fit with their professional skills and responsibilities. Flow sheet 1 was restructured to group the assessment sections by team member. Previously the occupational therapist and home care nurse received occasional referrals from physicians and the nurse practitioner for cognitive or functional assessment, but were not routinely involved in discussions about the diagnosis or early care planning with the patient and family.

#### Care management

In the first trials of the team-based approach, the assessment was conducted sequentially, starting with the physician or nurse practitioner in the clinic, followed by separate home-based assessments by the home care nurse and occupational therapist. Once completed, the physician or nurse practitioner arranged for a case conference (if indicated) with the patient, family, Team 1 members, and Alzheimer Society First Link Coordinator. However, because the sequential approach meant delays between provider assessments and prolonged the process, the team moved to a one-stop memory clinic model held in the PHC clinic, with the case conference at the end of the day. The conference was guided by the content in PC-DATA flow sheet 2 (diagnosis and initial management). Both flow sheet 2 and flow sheet 3 (monitoring and ongoing care) included checklists of best practices for providing initial and ongoing post-diagnostic support to the patient and family following the case conference. These guidelines were helpful but accessing three different flow sheets in the EMR was cumbersome. To improve accessibility, usability, and continuity, a combined flow sheet was created in the EMR that incorporated the content from the three original flow sheets.

#### Education/support for individual with dementia and caregivers

Sun Country is one of six health regions in Saskatchewan that has an Alzheimer Society Resource Centre and First Link Coordinator. The First Link program (McAiney *et al*., 2012) is aimed at connecting patients and families with education and supports beginning at diagnosis. To build on this strength, we added a link in the EMR visit flow sheet to provide easy access to the First Link referral form and the regional First Link Coordinator participated in the assessment and case conferences.

### Decision support

#### Standardized tools/guidelines and access to IT resources

These elements are discussed together because the PC-DATA flow sheet was embedded in the EMR and included links to all required tests, referral forms, and the RaDAR Handbook. The Handbook, compiled by Team 1, included screen shots of the adapted EMR flow sheet, the adapted PC-DATA education manual and original care pathway/algorithms, scripts developed by Team 1 for communicating the diagnosis and addressing driving issues, work standards for completing the EMR flow sheet and organizing the case conference, and Alzheimer Society educational resources for patients and families. A folder with paper copies of these resources was placed in each physician and nurse practitioner office and provided to all team members. The EMR was accessible by all team members except home care. The region had a limited number of licenses available for EMR access due to the cost but access was granted to the Team 1 home care nurse for the study.

### Specialist-to-provider support

#### Access to dementia specialists and formal dementia training for PHC providers

Patients in Sun Country have been eligible for referral to the RaDAR team’s Rural and Remote Memory Clinic in Saskatoon since its inception in 2004 (Morgan *et al*., 2009). However, the long travel distance and the clinic waitlist are barriers, as is the lack of capacity of RaDAR clinical members to provide individual case-based support outside the regular memory clinic referral process. The PC-DATA developer provided an educational session to Team 1 on best practices in dementia assessment, diagnosis, and management that underpinned the content of the visit flow sheets. RaDAR clinical specialists (neurologist and neuropsychologist) provided interactive educational sessions via telehealth videoconferencing from Saskatoon, on topics identified by Team 1. The researchers organized a presentation on driving assessment by the Saskatchewan Government Insurance Medical Review Unit. These sessions were well received but strategies for providing specialist-to-rural provider support require additional focused study. A recent seven-year research program award to the RaDAR team will support research to develop and evaluate strategies for remote case-based support and education by specialists to rural PHC providers. The limited number of dementia specialists in the province is unlikely to change in the future, and feasible and effective strategies for providing remote support and education are needed.

### Step 5: sustaining the intervention in first PHC team while scaling-up to second team

The strategies developed with Team 1 are not yet solidly integrated in routine practice. Our collaboration with the team is continuing so that we can provide ongoing support in addressing implementation challenges, and to help identify the barriers and enablers to sustainability over time. We are now implementing the Rural PHC model developed with Team 1 in a second team, which is located in a larger center in Sun Country Health Region. Team 2 was recommended by the regional steering committee because it was more stable in terms of physician retention compared to other teams in the region that were smaller and had experienced recent turnover. This setting provides an opportunity to identify adaptations to the Rural PHC Model to fit a larger team that also includes social workers. Before implementing the one-day clinic model in Team 2, a joint working group meeting was held with Team 1, and the Team 2 physician attended a Team 1 clinic. A third team, which serves three small rural communities, has just started implementation. Several Team 3 members attended a Team 2 clinic to facilitate their implementation planning. These strategies have supported communication, sharing of experiences, and trouble-shooting between teams, as recommended by earlier studies of team-based primary care networks (Misfeldt *et al*., 2017) and spreading innovations (Yano *et al*., 2012). The teams are holding one-day clinics every one to two months, with two new patients and their families assessed. Team-based follow-up visits will also be scheduled on clinic days.

### Evaluation of the rural PHC model

This paper describes the co-development of the Rural PHC Model for Dementia with a rural PHC team, to operationalize best practices in PHC for dementia in rural contexts. Now that a functional, feasible model has been achieved in one team and scaled-up to two additional teams, we are beginning to conduct formal evaluations of the model. Patient and family experiences with the one-day clinic will be assessed via telephone interview and mail-in questionnaire, to help us understand how this model compares with other specialist assessments, satisfaction with the team-based approach of the model, and how the model can be improved. Intervention effectiveness outcomes (eg, quality of patient care and costs associated with one-day clinics versus usual care) will be evaluated by chart review in intervention and non-intervention teams in the region. We will also be examining the experiences of the PHC team members in delivering collaborative care to rural patients and families before and after implementation of the Rural PHC Model for Dementia.

## Discussion

A number of key learnings have emerged from this research, which includes the completion of all five development/adaptation steps with PHC Team 1 and the beginning of the process to scale-up to additional PHC teams. The first is that relationship-building and maintenance is essential before, during, and after implementation. Developing and sustaining relationships at both the regional and PHC team level have been critical to maintaining momentum in the partnership and working through the challenges that are inevitable when designing practice changes. Our experience is consistent with the recommendation that partnerships within and across organization levels, including clinicians and senior leaders, are necessary for implementation and sustainability (Callahan *et al*., 2011; Yano *et al*., 2012). However, building and sustaining trusting, productive relationships takes time and effort that is often underestimated (Shalowitz *et al*., 2009; Brush *et al*., 2011; Rycroft-Malone *et al*., 2016). The use of technology such as videoconferences and teleconferences has helped to keep momentum going between in-person meetings, there is no substitute for regular face-to-face contact. When researchers are at a distance from partners, time and cost of travel are significant challenges to building and sustaining relationships.

The implementation process is an iterative one of trial and error, until workable solutions have been found and tested. The five-step development–adaptation framework used in this study was useful and effective, and is consistent with community-based participatory research and implementation science principles that emphasize co-development of innovations. As others have noted, collaborative implementation research requires researchers to be comfortable with uncertainty and messiness (Yano *et al*., 2012; Rycroft-Malone *et al*., 2016). We found that the steps in the framework are not strictly linear; once reached, each requires ongoing attention. Relationship-building, needs assessment/problem-solving, resolving applicability issues, and implementing and evaluating become continuous activities. This study involved a hybrid of developing rural-specific strategies to operationalize and implement known best practices in PHC for dementia (eg, team-based care) and adapting existing decision support tools to the rural context and EMR. The decision to adapt PC-DATA was made collaboratively with the PHC team, based on fit with needs identified by the PHC team. Moreover, important was the fact that the PC-DATA tools were developed from the Canadian consensus guidelines and had been well-received by family physicians in Ontario, and that the developer agreed to assist with adaptation to the rural Saskatchewan PHC team context.

The development–adaptation process itself is time-intensive for community partners and researchers. However, our experience endorses the view that approaches that support local health care systems to adapt and adopt new knowledge are more likely to result in successful implementation and sustainability than imposing rigid adherence to an existing protocol (Callahan *et al*., 2011; Richard *et al*., 2015). Health care providers working in rural PHC settings have many competing demands that make it difficult to find the time and energy for intervention research that requires behavior changes. The challenge of finding time and space to adapt and plan change (Callahan *et al*., 2011) was partly addressed by creating a smaller working group that was easier to co-ordinate than the full PHC team. The intervention developed with Team 1 introduced a collaborative approach with shared assessment and care planning. Adapting the context (Wallerstein and Duran, 2010; Aarons *et al*., 2012) by changing practice patterns and structures takes time, as it involves devising and testing ways new ways of working together. Building capacity for using and sustaining the intervention is key (Cabassa *et al*., 2014) but also requires a time commitment from researchers and partners.

Using community-based participatory research practices to co-develop innovations in rural settings far from researchers introduces additional challenges to the process. Although collaborative research is recommended as a strategy to address local research questions and needs in underserviced rural and remote communities, Ritchie *et al*. (2013) found that community-based participatory research principles were more difficult to follow in distant locations compared to those located closer to urban academic hubs. This ‘proximity paradox’ (Ritchie *et al*., 2013: 187) highlighted the need to develop flexible collaborative approaches that reflect the place and proximity of the collaborating partners. In our case the long distance to rural communities added time and cost, and overnight trips for the research team were often needed. Face-to-face meetings were held less frequently due to the feasibility of travel, particularly in winter months due to inclement weather. Another challenge to implementation and sustainability was the high rate of turnover among physicians in PHC Team 1, where all three physicians had completed their contracts and relocated by the end of the initial implementation. Recruitment and retention of physicians is a reality in many rural communities. A study of primary care networks in Alberta, Canada, found that remote and rural networks experienced more issues with workforce turnover and availability than urban networks (Misfeldt *et al*., 2017). These realities highlight the need for rural PHC models to build in strategies for sustainability that do not rely on the continuous presence of any one team member.

Finally, understanding the factors influencing both initial implementation and longer-term sustainability is key to developing strategies for spreading (horizontal sustainability) and sustaining/embedding the intervention in the system (vertical sustainability) (Peters *et al*., 2013). In the initial implementation stage interventions are vulnerable to all the forces at play in a complex context with many demands (Fixen *et al.*, 2005). Maintaining our partnerships with the teams will help us to understand what community partners need from researchers to sustain interventions in the short and long term, and the contextual factors influencing sustainability, which may differ from those influencing initial implementation. Israel *et al*. (1998) identified three key dimensions of sustainability of academic–community partnerships that are relevant to this study: sustaining relationships and commitments between partners; sustaining the knowledge and capacity of the partnership; and sustaining the funding, staff, program, and policy changes.

### Strengths and limitations

The paper reports on the initial development phase of a research program aimed at finding strategies for operationalizing accepted principles of PHC for dementia, in ways that are feasible and effective in rural settings. This research addresses an identified gap in evidence to inform successful implementation and dissemination of evidence-based dementia care in rural PHC (Lourida *et al*., 2017). We expect that this first iteration of the Rural PHC model will need further adaptations as it is refined over time and scaled-up to other teams. One purpose of the research is to identify the contextual factors relevant to each team and their community that necessitate adaptation. We will continue to adapt the intervention with other teams, and to evaluate outcomes, to accumulate generalizable knowledge about dementia care best practices that work in diverse rural PHC teams and contexts. Ongoing model development will include a focus on sex and gender differences in needs of people with dementia and their caregivers, and how strategies can be adapted to meet those needs. Scaling-up and sustaining evidence-based PHC innovations and improving access to appropriate care in rural regions are national and international priorities.

## Conclusions

The approach used in this study provides an example of how to bridge the gap between evidence-based practice and practice-based evidence (Yano *et al*., 2012). Callahan *et al*. (2011: 7) notes that ‘spontaneous adoption’ of PHC models for dementia is unlikely therefore we need to understand and address the complexity of the health care system. Rural patients and caregivers and the health care system will benefit if dementia can be appropriately diagnosed and managed close to home, resulting in high-quality dementia care and enhanced quality of life for rural people with dementia and caregivers.

## Supplementary material

For supplementary material accompanying this paper visit https://doi.org/10.1017/S1463423618000968. click here to view supplementary material
