# Supplementary material for: A five-step approach for developing and implementing a Rural Primary Health Care Model for Dementia: a community–academic partnership
Source: Prim Health Care Res Dev. 2019 May 15;20:e29. doi: 10.1017/S1463423618000968 (PMC6536750; doi:10.1017/S1463423618000968)
Supplement: Supplementary file 1 [file S1463423618000968sup001.docx]

**Supplementary Material**. Core and Adaptable Elements of the Primary Care-Dementia Assessment and Treatment Algorithm (PC-DATA)^TM^

**What are core and adaptable elements?**

Core elements “represent the intent, theory, and internal logic of the intervention (and) must be implement with fidelity to increase the likelihood (of) outcomes that are similar to those in the original research” (McKleroy et al. 2006, p. 67). Core elements “are, by definition, essential to achieving good outcomes for those targeted by the intervention. This is as true for modes of delivery and intervention settings as it is for intervention content” (Michie et al. 2009, p. 2). Intervention developers may assist with identifying expected effects since “very few evidence-based interventions have evidence on core elements available” (Jansen et al. 2013, p. 9).

Non-core or adaptable elements are “other secondary aspects of the program that are not directly linked to the program theory or the core mechanisms (and) may be adapted to meet the needs of different populations” (Lee et al. 2008, p. 293). Adaptations of non-core elements may include deletions/additions/modifications of program elements and changes in the delivery or cultural aspects of program elements. The target setting may also require adaptation to increase acceptance of the intervention (e.g., improving capacity, increasing resources).

**PC-DATA**^TM^ **elements**

Elements include materials (intervention content, techniques, instruments), delivery mode, Intensity (frequency, sequence) and duration (length of contact time), organizational structure, and target population.

Every element in PC-DATA, and its components, is considered to be either core or adaptable. It should be noted that while some materials may be core, their content may be adaptable. For instance, education slides may be essential to the PC-DATA intervention, but the exact content of the slides may be adaptable to suit the SK health system context.

The assessment of the core and adaptable elements was conducted by the PC-DATA developer (D. Seitz) and Rural Dementia Action Research team clinical experts and researchers,

| **PC-DATA^TM^ ELEMENT** | **CORE (C) vs.**  **ADAPTABLE (A)** |
| --- | --- |
| **Materials (intervention content, techniques, and instruments)** |  |
| 1. **Information session and needs assessment meeting (with PCP group)** | Core |
| 1. **Initial education session - Group-based PC-DATA tool implementation workshop (with PCP group)** | Core |
| 1. **Follow-up education session (with PCP group)** | Core |
| 1. **Dementia Care Manager** | Core |
| 4.1 Setting |  |
| - RN affiliated with Providence Care Geriatric Psychiatry Outreach Program (Queen’s, Kingston ON) | A |
| 4.2 Responsibilities |  |
| - facilitates application of PC-DATA and provides support to PCP | A |
| - accepts referrals from PCP (if patient screens positive or has cognitive complaints) | A |
| - arranges visits with patient and caregiver on-site or at home | A |
| - may assist with evaluation, caregiver support, service coordination | A |
| - reviews with geriatric psychiatrist as needed | A |
| 1. **Geriatric Psychiatrist** | Core |
| 5.1 Setting |  |
| - Providence Care – Mental Health Services, Kingston ON | A |
| 5.2 Responsibilities |  |
| - visits for complex patients | A |
| 1. **Education Slides (see Education Manual for slide topics)** | Core |
| 1. **Education Manual (accompanies the Education Slides)** | Core |
| 7.1 Evaluation | C |
| Why assess cognition in the elderly? | C |
| - Who should receive cognitive assessment? | C |
| - Which tests can be used for screening of cognitive changes? | C |
| - MMSE | C |
| - Mini-Cog | C |
| - Informant Self-Reports - Health Aging Brain Centre - Monitor (HABC-M) | C |
| - Informant Self-Reports - AD8 | C |
| - Informant Self-Reports - IQCODE 12 | C |
| Montreal Cognitive Assessment (MoCA) | C |
| History, physical examination, screening investigations | C |
| - Differential diagnosis of dementia | C |
| - History of Cognitive Changes | C |
| - Types of Dementia | C |
| - Medication Review and Anticholinergic medications | C |
| - Investigations (physical exam, screening bloodwork, and neuroimaging) | C |
| Neuropsychological Testing | C |
| Functional Assessment | C |
| - Functional Assessment Questionnaire (FAQ) | C |
| - Katz Activities of Daily Living (ADL) | C |
| Diagnosis of Dementia | C |
| Mild Cognitive Impairment | C |
| Normal Aging | C |
| Approach to Diagnostic Uncertainty | C |
| 7.2 Initial Management | C |
| Types of Dementia | C |
| Disclosure of Diagnosis | C |
| - Address Immediate Safety Concerns | C |
| Addressing Information Needs | C |
| Nonpharmacological Treatments | C |
| Pharmacological Treatments | C |
| - Cholinesterase Inhibitors | C |
| Cholinesterase Inhibitors Investigations and Contraindications | C |
| Dosing of Cholinesterase Inhibitors | C |
| Side-effects with Cholinesterase Inhibitors | C |
| Monitoring Response to Therapy | C |
| Memantine (Ebixa) | C |
| Incidence and Management of Side-effects with Memantine | C |
| Memantine Dosing | C |
| Discontinuing Dementia Medications | C |
| Other Pharmacological Treatments | C |
| 7.3 Ongoing Assessment and Monitoring | C |
| Ethicolegal Recommendations | C |
| - Capacity | C |
| - Understanding | C |
| - Appreciate | C |
| - Dementia and Capacity | C |
| - Capacity and Degree of Cognitive Impairment | C |
| - Capacity | C |
| - Aid to Capacity Evaluation | C |
| Capacity | C |
| - Capacity Resources | C |
| Contact Public Guardian/Trustee | C |
| Additional Safety Concerns | C |
| Driving | C |
| - Cognition and Driving | C |
| - Driving Evaluation | C |
| - Driving Resources | C |
| - Information on Driving for Patients | C |
| Reporting Requirements | C |
| Behavioural Symptoms in Dementia | C |
| - Prevalence of Neuropsychiatric Symptoms | C |
| - Prevalence of NPS in Long Term Care | C |
| - Assessment of Behavioural Symptoms | C |
| Management of Behavioural Symptoms | C |
| - Caregiver Supports for Behavioural Symptoms | C |
| - Monitoring Caregiver Stress | C |
| - Pharmacological Treatment | C |
| - Agitation, Aggression, and Psychosis | C |
| - Depression or Anxiety Symptoms | C |
| - Sleep Changes | C |
| - Monitoring Behavioural Symptoms | C |
| 7.4 Using PC-DATA Website | C |
| Contact Dementia Care Manager | C |
| 7.5 Contact Information | C |
|  |  |
| 1. **Flow sheets (evaluation, initial management, ongoing assessment and monitoring)** | Core |
| 8.1 Evaluation | C |
| Demographic Data | A |
| Presenting Complaint | A |
| History of Cognitive Changes | A |
| Cognitive Testing | A |
| Medical History | A |
| Medications | A |
| Past Psychiatric History | A |
| Family History | A |
| Physical Exam | A |
| Bloodwork, EKG, Imaging | A |
| Functional Assessment | A |
| Diagnosis | C |
| Follow-Up Plans | A |
| 8.2 Initial Management | C |
| Determine Type of Dementia | A |
| Disclosure of Diagnosis | A |
| Information about Dementia and Services | A |
| Nonpharmacological Management | A |
| Discussion of Treatment with Cholinesterase Inhibitors | A |
| Initiating Treatment with Cholinesterase Inhibitors | A |
| Adverse Events with Cholinesterase Inhibitors | A |
| Preventing Side-effects | A |
| Monitoring Treatment Benefits and Emergent Side-effect with Cholinesterase Inhibitor | A |
| Discussion of Treatment with Memantine | A |
| Adverse Events with Memantine | A |
| Follow-Up Plans | A |
| 8.3 Ongoing Assessment and Monitoring | C |
| Monitoring of Cognition | A |
| Evaluation of Capacity | A |
| Behavioural and Psychological Symptoms of Dementia | A |
| Household Safety | A |
| Driving Safety | A |
| Follow-Up Plans | A |
|  |  |
| 1. **Algorithms (evaluation, initial management, ongoing assessment and monitoring) ^a^** | Core |
| 9.1 Evaluation Algorithm | C |
| Brief Cognitive Tests | C |
| - Tool - Mini-cog | A |
| - Tool - GPCOG | A |
| - Tool - AD8 | A |
| - Tool - IQCODE (short) | A |
| - Tool - IQCODE (long) | A |
| - Resource - MMSE scores in the general population | A |
| Test with MoCA | C |
| - Tool - MoCA | C |
| - Resource - MOCA | C |
| MoCA Test Results | C |
| - Tool - MoCA norms in the General Population | C |
| Screening Investigations | C |
| - Tool - History of cognitive changes | C |
| - Tool - Differential diagnosis in dementia | C |
| - Tool - Physical exam, bloodwork, imaging | C |
| - Tool - Anticholinergic medications | C |
| Assessment of Functional Impairment | C |
| - Tool - Functional Activities Questionnaire (FAQ) | A |
| - Tool - Katz Activities of Daily Living (ADL) | A |
| Diagnosis: Dementia | C |
| - Tool - Normal Aging and Risk Factor Management | C |
| Diagnosis: Mild Cognitive Impairment | A |
| - Tool - Mild Cognitive Impairment | A |
| 9.2 Initial Management Algorithm | C |
| Determine type of dementia | A |
| - Tool - Types of Dementia | A |
| - Tool - Websites for information (Alzheimer.ca, Dementiaguide.ca, Alzheimerbc.org) | A |
| - Tool - Supports and services | C |
| - Tool - Nonpharmacological treatments | A |
| - Resource - Nonpharmacological treatments (Alzheimer Society) | A |
| Starting a Cholinesterase Inhibitor | C |
| - Tool - Information on Cholinesterase inhibitors | A |
| - Resource - Websites for information (Donepezil, Rivastigmine, Galantamine) | A |
| Cholinesterase Inhibitors Prescreening and Side-effects | A |
| - Tool - CheEI contraindications and investigations | A |
| Dosing of Cholinesterase Inhibitors | A |
| - Tool - Dosing of Cholinesterase Inhibitors | A |
| Assessment of Treatment Response | A |
| - Tool - Incidence and management of side-effects of cholinesterase inhibitors | A |
| Discussion of Memantine | A |
| - Tool - Memantine | A |
| - Resource - Memantine | A |
| Memantine Prescreening and Side-Effects | A |
| - Tool - Incidence and management of side-effects with memantine | A |
| Dosing of Memantine | A |
| - Tool - Dosing of Memantine | A |
| - Resource - Memantine | A |
| 9.3 Ongoing Assessment and Monitoring Algorithm | C |
| Assessment of Capacity | A |
| - Tool - Capacity table | A |
| - Tool - Capacity | A |
| - Resource - Advocacy Centre for the Elderly | A |
| - Resource - Aid to Capacity Evaluation (ACE Tool) | A |
| Driving Assessment | A |
| - Resource - Driving and Dementia Toolkit | A |
| - Resource - Ministry of Transportation | A |
| - Resource - CANDrive | A |
| Safety Concerns at Home | A |
| - Resource - Additional Safety Concerns | A |
| Behavioural Concerns | A |
| - Tool - Prevalence of Neuropsychiatric Symptoms | A |
| - Tool - Assessment of Behavioural Symptoms | A |
| - Tool - Neuropsychiatric Inventory | A |
| - Resource - Reducing Caregiver Stress | A |
|  |  |
| 1. **Healthy Aging Brain Centre (HABC) Monitor** | Adaptable |
|  |  |
| 1. **OHIP Codes and Procedures for dementia** | Adaptable |
|  |  |
| 1. **Services in Ontario** | Adaptable |
|  |  |
| 1. **Behavioural Changes in Older Adults** | Adaptable |
|  |  |
| **Delivery mode ^b^** |  |
| 1. Information session and needs assessment meeting with PCP group |  |
| - in-person | A |
| 2. Initial education session - Group-based PC-DATA tool implementation workshop |  |
| - in-person | A |
| 3. Follow-up education session |  |
| - in-person | A |
| 4. Dementia Care Manager (facilitates PC-DATA tool application and provides support) |  |
| - in-person | A |
| - by phone | A |
| 5. Geriatric Psychiatrist |  |
| - in-person | A |
| - by phone | A |
| 6. Education Slides |  |
| - in-person powerpoint | A |
| - electronic (website) | A |
| 7. Education Manual |  |
| - electronic (website) | A |
| 8. Flow sheets (evaluation, initial management, ongoing assessment and monitoring) |  |
| - electronic (website) | A |
| 9. Algorithms (evaluation, initial management, ongoing assessment and monitoring) |  |
| - electronic (website) | A |
|  |  |
| **Intensity (frequency, sequence) and duration (length of contact time per visit)** |  |
| 1. Information session and needs assessment meeting with PCP group (to tailor intervention to local practice) |  |
| - Intensity = 1 time minimum, prior to implementation of intervention | C |
| - Duration = 1 hour | A |
| 2. Initial education session - Group-based PC-DATA tool implementation workshop |  |
| - Intensity = 1 time minimum, at initiation of intervention | C |
| - Duration = 1 to 2 hrs | A |
| 3. Follow-up education session |  |
| - Intensity = 1 time minimum, at study mid-point | C |
| - Duration = 1 hr | A |
| 4. Dementia Care Manager (facilitates PC-DATA tool application and provides support) |  |
| - Intensity = depends on PCP, for duration of intervention | A |
| - Duration = variable | A |
| 5. Geriatric Psychiatrist |  |
| - Intensity = depends on PCP, for duration of intervention | A |
| - Duration = variable | A |
| 6. Education Slides |  |
| - Intensity = always available, introduced at initial education session? | C |
| - Duration = n/a | n/a |
| 7. Education Manual |  |
| - Intensity = always available, introduced at initial education session? | C |
| - Duration = n/a | n/a |
| 8. Flow sheets (evaluation, initial management, ongoing assessment and monitoring) |  |
| - Intensity = always available, introduced at initial education session? | C |
| - Duration = n/a | n/a |
| 9. Algorithms (evaluation, initial management, ongoing assessment and monitoring) |  |
| - Intensity = always available, introduced at initial education session? | C |
| - Duration = n/a | n/a |
|  |  |
| **Organizational Structure** |  |
| 1. University researcher(s) deliver the intervention | A |
| 2. Dementia specialist(s) deliver the intervention | C |
| **Target population** |  |
| 1. Primary care providers | C |
| - family physicians | A |
| - nurses | A |
| - social workers | A |
| 2. Primary care groups (e.g., family health teams, family health organizations) | A |
| 3. Southeastern Ontario | A |

**References**

**Jansen S., Haveman-Nies A., Duijzer G., Beek J., Hiddink G.,** and **Feskens E.** 2013: Adapting the SLIM diabetes prevention intervention to a Dutch real-life setting: joint decision making by science and practice. *BMC Public Health* 13:457.

**Lee S., Altschul I.,** and **Mowbray C.** 2008: Using planned adaptation to implement evidence-based programs with new populations. *American Journal of Community Psychology* 41:290–303.

**McKleroy VS, Galbraith JS, Cummings B, Jones P, Harshbarger C, Collins C, Gelaude D, Carey JW,** and **the ADAPT team**. 2006: Adapting evidence-based behavioural interventions for new settings and target populations. *AIDS Education and Prevention* 18, 59-73.

**Michie S., Fixsen D., Grimshaw J.,** and **Eccles M.** 2009: Specifying and reporting complex behavior change interventions: the need for a scientific method (editorial). *Implementation Science* 4:40.
